# Achieving remarkable mechanochromism and white-light emission with thermally activated delayed fluorescence through the molecular heredity principle†
†Electronic supplementary information (ESI) available: Details of the synthesis; structural information for the compounds (NMR, elemental analysis and mass spectra); Fig. S1–S28. CCDC 1421264–1421266. For ESI and crystallographic data in CIF or other electronic format see DOI: 10.1039/c5sc04155d


**DOI:** 10.1039/c5sc04155d

**Published:** 2016-01-11

**Authors:** Bingjia Xu, Yingxiao Mu, Zhu Mao, Zongliang Xie, Haozhong Wu, Yi Zhang, Chongjun Jin, Zhenguo Chi, Siwei Liu, Jiarui Xu, Yuan-Chun Wu, Po-Yen Lu, Alan Lien, Martin R. Bryce

**Affiliations:** a PCFM Lab , GD HPPC Lab , Guangdong Engineering Technology Research Center for High-performance Organic and Polymer Photoelectric Functional Films , State Key Laboratory of Optoelectronic Material and Technologies , School of Chemistry and Chemical Engineering , Sun Yat-sen University , Guangzhou 510275 , China . Email: ceszy@mail.sysu.edu.cn ; Email: chizhg@mail.sysu.edu.cn ; Fax: +86 20 84112222 ; Tel: +86 20 84112712; b State Key Laboratory of Optoelectronic Material and Technologies , School of Physics and Engineering , Sun Yat-sen University , Guangzhou 510275 , China; c Shenzhen China Star Optoelectronics Technology Co., Ltd , Shenzhen 518107 , China; d Department of Chemistry , Durham University , DH1 3LE , UK . Email: m.r.bryce@durham.ac.uk

## Abstract

Achieving high contrast mechanochromism (Δ*λ*_em,max_ > 100 nm) and white-light emission under mild conditions from a single compound with a simple structure is a great challenge. Herein, we report a novel dual-emissive compound, namely SCP, with an asymmetric molecular structure that fully inherits the photophysical properties of the parent molecules SC_2_ and SP_2_. SCP shows high contrast, linearly tunable mechanochromism and bright white-light emission arising from a combination of traditional fluorescence and thermally activated delayed fluorescence (TADF). The origin of the dual-emission for SCP was demonstrated based on the analysis of the white-emitting single crystals. In addition, a mechanism of luminochromism for SCP driven by the application of mechanical force is proposed. These observations present a rational design strategy for the development of high performance multi-functional materials for white-light emission.

## Abstract 2

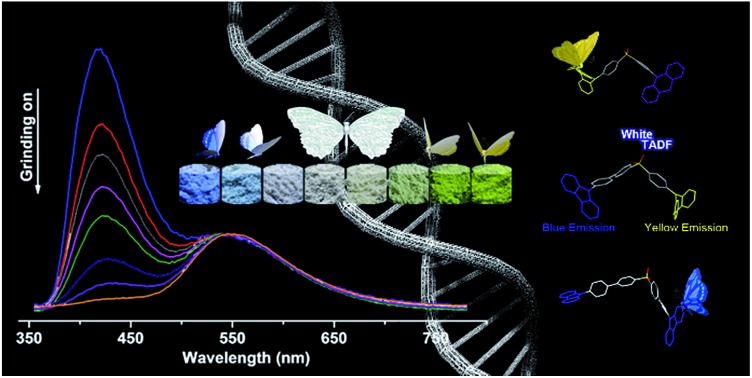
An asymmetric molecule created by molecular heredity exhibits remarkable mechanochromism and white-light emission.

## Introduction

Heredity, a universal phenomenon in nature, is the process by which an offspring acquires or becomes predisposed to the features or characteristics of its parents. Through heredity, distinctive capacities of the species are transmitted from ancestor to descendant and, simultaneously, variations exhibited by individuals can cause the species to evolve through natural selection. In a biological context, heredity plays a key role in improving the essential capacities or achieving new useful skills for a species to adapt to a new living environment.^1^ It would be very interesting to utilize such a principle for molecular design to enhance the development of new materials, for instance, creating novel dyes with remarkable mechanochromism and white-light emission.

Organic mechanochromic luminophores have attracted tremendous attention due to their promising applications as pressure sensors, fluorescent switches and optical devices.^2^ To date, despite the active search for high performance mechanochromic materials, most of the reported compounds exhibit merely dual-colored alteration with a limited emission switching range (mostly, Δ*λ*_em,max_ < 100 nm).2b,^3^ Organic dyes with tunable and remarkable luminochromism which respond to mechanical force are scarce and, so far, only a few examples have been reported, even when sophisticated fabrication processing or compression under high-pressure was employed.2a,2c,^4^ Noticeably, although mechanochromic materials with various colors have been documented, single compounds showing white-light emission resulting from a mechanical stimulus are rare.^5^ As materials for practical illumination and device displays, white-emitting dyes have attracted increasing interest in the last few decades.^6^ However, since the majority of luminophores obey Kasha's rule, producing luminescence in appreciable yield only from the lowest excited state, it is unusual for a single organic compound to emit either two (blue/yellow) or three (blue/green/red) colors covering the entire visible spectral window from 400 to 700 nm.^7^ In addition, energy transfer often occurs within a single molecule, leading to the dominant emission color corresponding to the lowest energy species. These intrinsic limitations impede the development of white-emitting materials. Therefore, achieving high contrast mechanochromism and white-light emission under mild conditions from small molecules with simple structures is a great challenge in synthetic chemistry, photochemistry and photophysics.

Recently, a white-light compound (4-(10*H*-phenothiazin-10-yl)phenyl)(4-(9*H*-carbazol-9-yl)phenyl)methanone (OPC, Fig. S1a†) with CIE_*x*,*y*_ coordinate of (0.35, 0.35) has been created by our group through the molecular heredity principle.^8^ This work thus offers the possibility for the development of white-emitting mechanochromic materials by selecting two mechanochromic compounds with blue emission and yellow emission, respectively, as parents of an asymmetric offspring dye. However, the mechanism for the generation of white-light emission of OPC remains unclear owing to the lack of dual-emissive single crystals. Hence, clarifying the origin of dual-emission is crucial for refining the molecular heredity strategy to obtain high performance multi-functional white-emitting dyes.

**Fig. 1:**
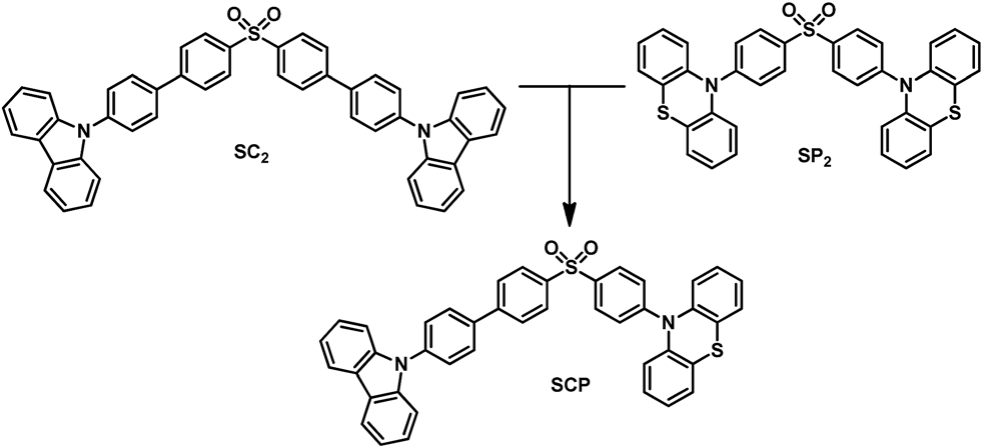
Molecular structure of the compounds.

In this article, we exploit the strategy of molecular heredity in developing a high contrast mechanochromic luminophore with white-light emission and the origin of the compound's dual-emission is established. The symmetric compounds SC_2_ and SP_2_ (Fig. 1) are the parent molecules. SC_2_ is a dye with impressive deep blue emission, whereas SP_2_ yields greenish-yellow TADF.^9^ Organic TADF compounds are regarded as the next-generation luminescent materials after fluorescent and phosphorescent luminophores due to their triplet harvesting features, which can theoretically convert electricity into light with an internal electroluminescence quantum efficiency of 100%.^10^ Consequently, considerable effort has been devoted to the synthesis and characterization of efficient TADF materials, particularly those with white-emitting properties in the non-doped solid state.6a,^11^ It would be an advantage if the triplet harvesting properties could be transmitted to derivatives directly from one or more reported TADF moieties by means of molecular heredity. Such a convenient approach can largely avoid the complex molecular design protocols for functional white-emitting TADF compounds, which would be potential candidates for efficient and multi-responsive photoelectric devices. Further study reveals that both SC_2_ and SP_2_ are mechanochromic luminophores. Based on this meticulous design, their offspring, namely SCP, is thus expected to inherit the two colors of deep blue and greenish-yellow which could combine to generate white-light emission with TADF, and to show enhanced mechanochromic properties.

## Results and discussion

**Fig. 2:**
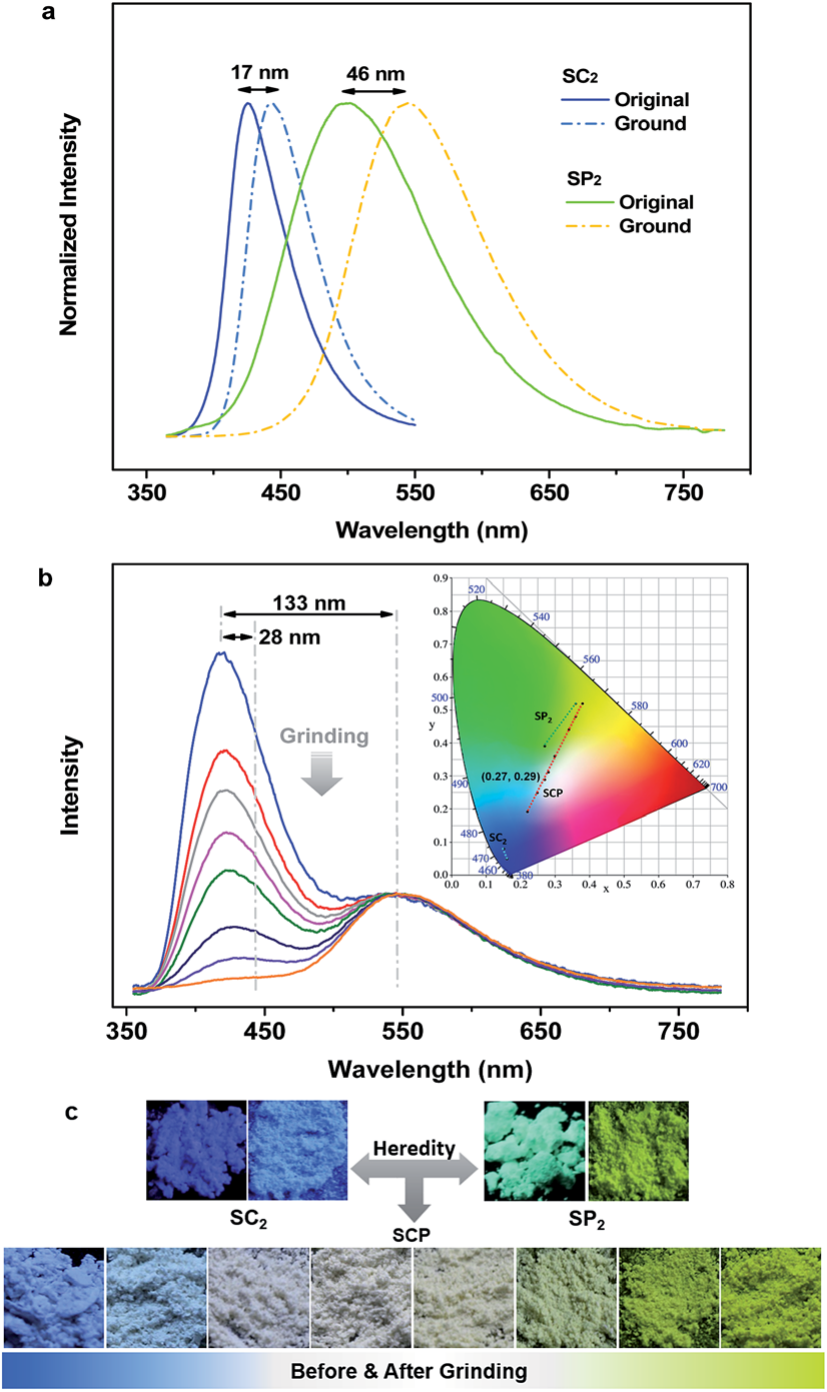
PL spectra and fluorescent images of the powders of the three compounds in solid state. (a) PL spectra of SC_2_ and SP_2_ in solid state. (b) Changes of PL spectra of SCP under grinding. (c) Images of the compounds taken under illumination by 365 nm UV light.

To specify the hereditary characteristics of this family, the photophysical properties of the parent molecules in the solid state were investigated. As depicted in Fig. 2a, the main peaks in the photoluminescence (PL) spectra for pristine SC_2_ and SP_2_ are centered at 426 nm [*Φ*_F,s_ = 0.30] and 500 nm [*Φ*_F,s_ = 0.21], respectively. The short lifetime of 5 ns determined by the transient PL decay measured in air indicated that SC_2_ is a normal fluorescent emitter (Fig. S2a†). However, for SP_2_, both prompt and delayed components with lifetimes of 7 ns and 28 μs, respectively, were observed in the emission decay transient (Fig. S2b†), thus verifying its TADF character.^12^ By grinding with a pestle or shearing with a spatula, the PL spectrum of pristine SC_2_ shifted by 17 nm to 443 nm. Compound SP_2_ displayed a larger bathochromic shift in emission (46 nm) under the stimulus of mechanical force, changing from an initial green into yellow light at *λ*_max_ 546 nm. The CIE_*x*,*y*_ chromaticity coordinates of SC_2_ before and after grinding are (0.16, 0.05) and (0.15, 0.08), whereas those of SP_2_ are (0.26, 0.38) and (0.37, 0.53) (Fig. 2b). These results represent only mediocre mechanofluorochromic performance for the two parent molecules.

**Fig. 3:**
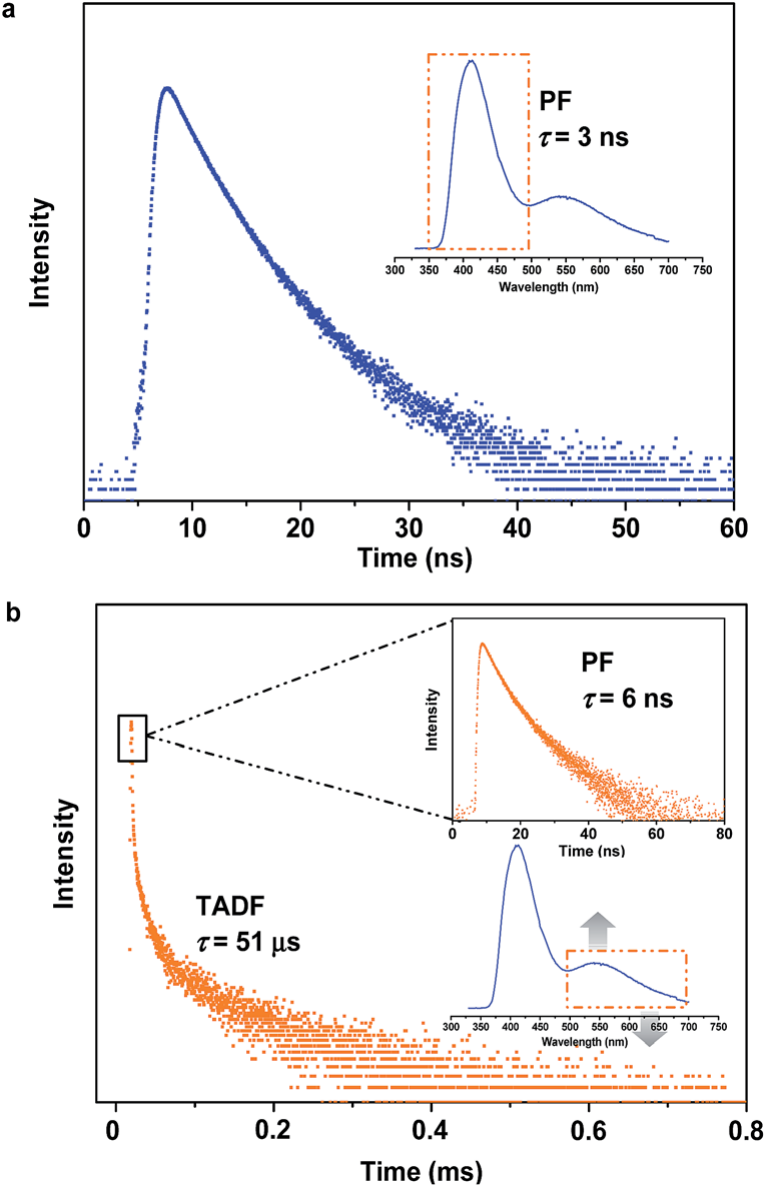
Emission decay curves of SCP. (a) For emission in the blue-light region. (b) For emission in the yellow-light region.

As expected, the as-prepared unsymmetrical SCP showed an intense dual-emission [*Φ*_F,s_ = 0.42] peaking at 415 nm and 545 nm in the solid state (Fig. 2b) which is close to the pristine sample of SC_2_ and the ground powder of SP_2_, respectively. The room temperature transient PL decay curves of these two peaks are shown in Fig. 3. The emission band in the deep blue region exhibited a prompt decay with a lifetime of 3 ns, typical of normal prompt fluorescence. By contrast, the yellow emission band displayed two-component decays, namely, a fast component with a lifetime of 6 ns and a slow component with a lifetime of 51 μs, which are assigned to the prompt and delayed fluorescence decays, respectively. The TADF characteristics of the 545 nm peak of SCP were also confirmed by the temperature dependent emission decay of the delayed component (Fig. S3†), which showed a continuous increase of lifetime from 6 μs at 77 K to 107 μs at 250 K, and a decrease at 300 K (59 μs).^13^ Further evidence for TADF was provided by the oxygen-sensitive PL spectra (Fig. S4†), indicating that the T_1_ excitons of SCP are deactivated by triplet oxygen in solid state. These findings unambiguously demonstrate that the essential photophysical properties of both SC_2_ and SP_2_, including the TADF character, are fully inherited by SCP. In addition, we reasoned that SCP would probably show aggregation-induced delayed fluorescence enhancement (AIDFE) by taking account of its asymmetric structure.^8^,9b To identify this feature, a tetrahydrofuran (THF) solution of SCP was titrated with water and the change in PL was monitored. As depicted in Fig. S5,† SCP exhibited an extremely weak dual-emission in THF solution, where it was well dissolved. However, when 95% (v/v) of water was added, a strong luminescence was observed and the corresponding intensities increased by up to ∼8 times as compared to those at 0% water fraction. Mie scattering effects which emerged in the UV-visible absorption profiles of SCP revealed that nanoaggregates of this dual-emissive luminophore were formed in the mixtures with high water contents. Evidently, SCP is AIDFE-active.^14^ The enhancement of TADF also illustrated that efficient reverse intersystem crossing (RISC) is maintained from solution to solid state in this molecule, which would be beneficial for the harvesting of triplet excitons in non-doped systems.

Fluorescence spectroscopy was also used to evaluate the influence of applied pressure on the dual-luminescence of SCP. As noted above, the *λ*_max_ for TADF of original SCP (545 nm) corresponded to the ground sample of SP_2_ (546 nm). Consequently, the original SCP should completely lose its mechanochromic activity in the yellow-light region according to the heredity principle. Indeed, the yellow emission band of SCP exhibited essentially no change in wavelength (*λ*_max_ = 548 nm) under applied mechanical force (Fig. 2b). However, in contrast to the TADF emission, the PL maximum of pristine SCP in the deep blue region gradually shifted from 415 nm to 443 nm, in agreement with the emission of ground SC_2_ (443 nm). Accompanying this change, the PL intensity of SCP in the blue-emitting region was reduced and the ratios between the deep blue- and the yellow-emission bands were reversed, thus leading to a notable bathochromic shift in the emission maximum of the whole spectrum by up to 133 nm. This represents much higher contrast mechanochromism for SCP in comparison with SC_2_ and SP_2_.

The pristine SCP powder is crystalline, as shown by the sharp and intense reflection peaks in its X-ray diffraction (XRD) pattern (Fig. S6a†). After applying mechanical force, the diffractograms of the samples changed very little, except for the well ground sample. Similar results were obtained by differential scanning calorimetry (DSC), although additional exothermal peaks around 129 °C and 159 °C were observed in the thermogram of the well-ground powder (Fig. S6b†). These results imply that the vivid mechanochromism for SCP mainly originates from local conformational changes of the molecules. Intriguingly, when the preceding emission changes were converted to the CIE_*x*,*y*_ chromaticity coordinates, all the points lie on a straight line with an excellent correlation coefficient (0.9996). This demonstrates that the color of SCP is linearly tunable from (0.22, 0.19) to (0.38, 0.52) under the mild treatment of hand grinding. Noticeably, as a consequence of the parent molecules of SC_2_ and SP_2_ endowing SCP with the deep-blue and yellow emission features and mechanochromic properties, SCP shows bright white emission with tunable CIE_*x*,*y*_ coordinates by applying the appropriate mechanical stimulus on the original sample (Fig. 2b) or by controlling the fuming time of a ground sample in the vapor of dichloromethane (Fig. S7†). A white-emitting powder with CIE_*x*,*y*_ coordinates of (0.27, 0.29) located on the fitting line could also be obtained simply by adding a concentrated SCP/THF solution into ethanol under the action of ultrasound. These observations thus clearly demonstrate that creating an asymmetric molecule through the molecular heredity principle is a viable strategy to achieve remarkable mechanochromism and efficient white-light emission with triplet harvesting. As previously reported, 10-(4-((4-(9*H*-carbazol-9-yl)phenyl)sulfonyl) phenyl)-10*H*-phenothiazine (SFPC, Fig. S1b†), which contains one fewer benzene rings than SCP, also exhibits dual-emission.^8^ However, white-light emission could not be observed in SFPC because of the poor performance of the blue-emitting component. This demonstrates the importance of the precise selection of the parent molecules.

**Fig. 4:**
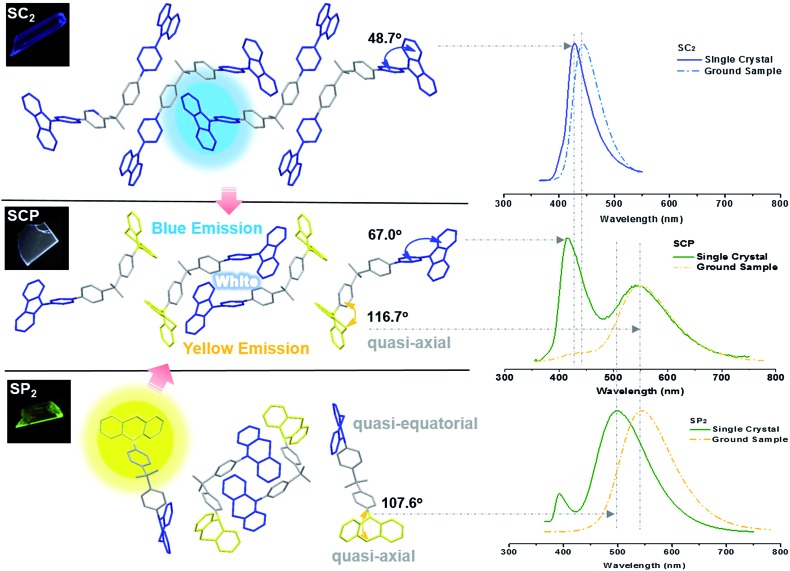
Molecular packing of the compounds in single crystals structures and PL spectra of the single crystals and ground samples. Insets are the fluorescence images of the three single crystals under illumination by 365 nm UV light.

Further study has focused on the origin of dual-emission for SCP. A single crystal X-ray investigation was thus performed for ‘gene detection’ within the whole ‘family’. Single crystals of SC_2_ and SP_2_ emitted intense deep blue and green light at *λ*_max_ 428 and 498 nm, respectively (Fig. S8a†). These values are close to those of their as-prepared powders (426 and 500 nm, respectively). The main peaks of the simulated XRD patterns from single crystal data of the two compounds also agreed well with those in the experimental patterns obtained from their original powders (Fig. S8b†), suggesting that the molecular packing modes for most of the molecules of these two compounds in solid powder are similar to the corresponding single crystals. Both the solid powder and the single crystals of SP_2_ are dual-emissive with an additional weak band located at around 392 nm (lifetime: 2 ns, Fig. S9†). Such a distinction in the number of emission bands between SP_2_ and SC_2_ can be ascribed to the different symmetries of their molecular conformations. As compared to the identical carbazoles in SC_2_ (Fig. 4), the two phenothiazine moieties in one SP_2_ molecule adopt ‘quasi-equatorial’ and ‘quasi-axial’ conformations in the single crystal structure. In other words, SP_2_ acts as a special asymmetric compound in the solid state, which is different from the results reported previously.9*b*

**Fig. 5:**
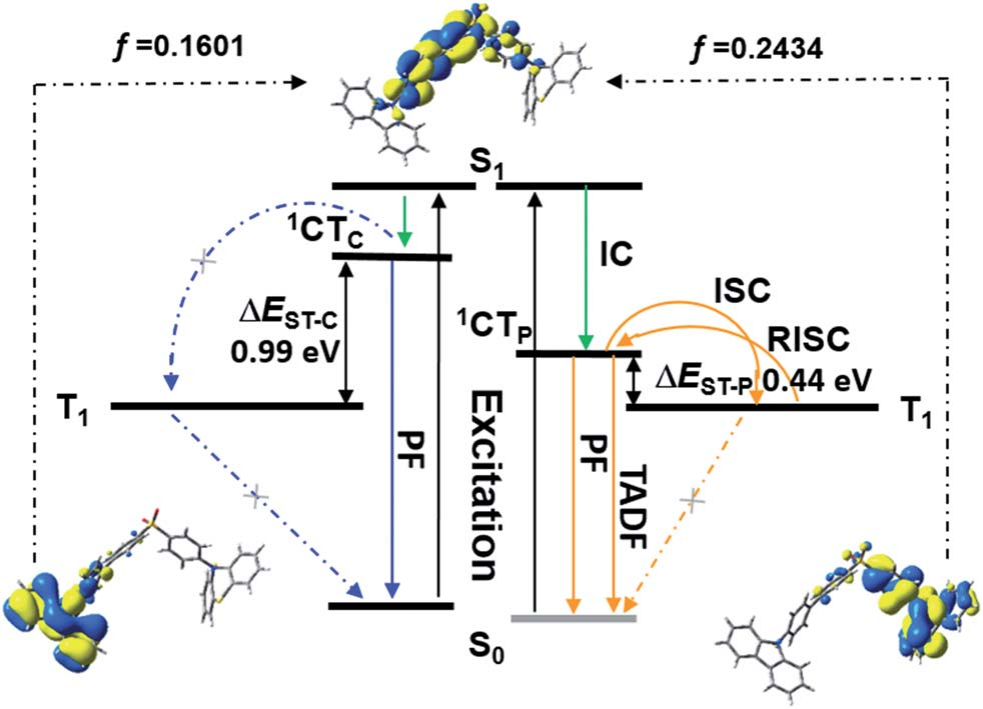
Electronic transitions and the corresponding decays in SCP.

In the case of SCP, white-emitting single crystals were achieved unexpectedly. These crystals provide a unique prototype to investigate the origin of dual-emission for SCP. As indicated in Fig. 4, the phenylcarbazole from SC_2_ and the ‘quasi-axial’ phenothiazine from SP_2_ have been passed on to SCP and serve as ‘gene segments’ to ‘express’ both deep blue and a yellow emission in the PL spectrum of SCP. The corresponding PL spectrum was almost superimposable on that of the as-prepared powder precipitated from THF/ethanol (Fig. S10a†), suggesting that the white-emitting powder of SCP probably adopts the same molecular arrangement as that of the white-emitting single crystals. Further evidence for this standpoint is provided by the overlap of the simulated XRD pattern from the single crystal data and the experimental pattern of the solid powder for SCP (Fig. S10b†). Meanwhile, only one kind of molecular conformation of SCP was observed in this white-emitting single crystal, suggesting that the dual-emission probably comes from the same molecular conformation of SCP, which is different from a previous hypothesis.^8^ Furthermore, emission from excimers could be ruled out, because no typical π–π stacking was observed in the crystal structure (Fig. S11†). Therefore, the dual-emission of SCP is most likely from two different radiative decays in a single molecule. Density functional theory at B3LYP/6-31+G(d,p) level was used to calculate the energy levels and the electronic transitions in SCP based on its ground state geometry in the single crystal structure. The results show that two electronic transitions from the occupied orbitals delocalized over the carbazole (highest occupied molecular orbital, HOMO; oscillator strength *f* = 0.1601) and phenothiazine (HOMO – 1, *f* = 0.2434) moieties to the diphenylsulfone (lowest unoccupied molecular orbital, LUMO) unit make major contributions to the excited states of SCP (Fig. 5). In other words, under excitation by UV light, there are two independent intramolecular charge-transfer (ICT) transitions from the carbazole and phenothiazine, respectively, to the diphenylsulfone acceptor unit in SCP. Accordingly, a shoulder band at around 360 nm and a maximum centered at 349 nm were observed in the solid state UV-visible spectrum for the white powder of SCP (Fig. S12†), which should be undoubtedly assigned to the ^1^CT transitions of the carbazole and phenothiazine moieties. Noticeably, the absorptions of the carbazole and phenothiazine moieties in SCP are close to each other and the absorption spectrum of SCP intersects with the PL spectra of both the white-emitting powder and the single crystals in the blue light region. These results indicate that radiative decays disobeying Kasha's rule and energy transfer from the blue light emission to the yellow-emitting band would probably occur in this asymmetric system. However, the strength of energy transfer in SCP seems to be controllable by tuning the aggregation state, resulting in the existence of dual-emission with appropriate relative intensity for white-light generation.

Furthermore, in different solutions, both of the two broad and structureless emission bands of SCP exhibit positive solvatochromism (Fig. S13†). The deep blue and the yellow emissions of SCP can be assigned to the radiative decays of the excited ^1^CT states of the carbazole and phenothiazine moieties, respectively.7a,^15^ The phosphorescence spectrum of SCP in toluene at 77 K is well resolved and shows the characteristic vibrational structure for phenothiazine (Fig. S14†), indicating that its T_1_ state has ^3^ππ* character located mostly on the electron donating phenothiazine moiety. As reported previously, the energy level of a CT state without any vibronic structure can be estimated from the onset of its broad emission band, while that of a ^3^ππ* state can be identified from the highest energy peak of the emission.11*c* From Fig. S14,† the energy gap (Δ*E*_ST_) between the ^1^CT state of phenothiazine in SCP and the T_1_ state of the molecule was determined to be 0.44 eV, which is much smaller than that between the ^1^CT state of carbazole and the T_1_ state of SCP (0.99 eV). The relatively small Δ*E*_ST_ value suggests a potentially high RISC, thus causing the TADF of the yellow emission band in SCP.11c,^16^

Additionally, the bend angle between the ‘quasi-axial’ phenothiazine and the neighboring phenyl ring in the single crystal structure of SCP is 116.7° (Fig. 4), which is larger than the corresponding angle in SP_2_ (107.6°). As a result, the TADF maximum of SCP (*λ*_max_ = 545 nm) is bathochromically shifted in comparison with the parent molecule SP_2_ (500 nm). On the contrary, as compared to SC_2_, the emission of SCP in the blue-light region (*λ*_max_ = 419 nm) showed a slight hypsochromic shift, which can be attributed to the larger dihedral angle *θ* between the carbazole and the adjacent phenyl ring (*θ* = 67.0° for SCP and *θ* = 48.7° for SC_2_). This different behavior is further evidence for the different photophysical processes (traditional fluorescence and TADF) which lead to the dual emission. Considering that the blue emission band of ground SCP is in agreement with that of the ground sample of SC_2_ and the UV-visible absorption spectrum of the well-ground SCP showed larger overlap with the corresponding PL spectrum in comparison with the pristine samples (Fig. S12†), the luminochromism of SCP driven by the mechanical force is believed to be correlated with the conformational planarization of the phenylcarbazole moiety and the stronger energy transfer from the blue light emission to the yellow-emitting band, leading to the red shift of the emission peak and the decrease of PL intensity for SCP in the blue-emitting region.

## Conclusions

The asymmetric compound SCP has been synthesized and shown to exhibit remarkable and linearly tunable mechanochromism and bright white-light emission with TADF by fully inheriting the photophysical properties of the parent molecules SC_2_ and SP_2_. The deep blue and the yellow dual-emission of SCP can be assigned to two independent radiative decays of the excited ^1^CT states of the carbazole and phenothiazine moieties, respectively. In addition, it is proposed that the mechanism of luminochromism for SCP driven by the mechanical force correlates with the conformational planarization of the phenylcarbazole moiety and the stronger energy transfer from the blue light emission to the yellow-emitting band. Such unusual observations have demonstrated that creating asymmetric molecules following the principle of molecular heredity holds promise as a strategy for the development of high performance functional materials. Further study will focus on the application of this mechanochromic white-emitting material in pressure sensing and optical recording.

## Supplementary Material

Supplementary information

Crystal structure data
