# Supplementary material for: Achieving remarkable mechanochromism and white-light emission with thermally activated delayed fluorescence through the molecular heredity principle
Source: Chem Sci. 2016 Jan 11;7(3):2201–6. doi: 10.1039/c5sc04155d (PMC5975939; doi:10.1039/c5sc04155d)
Supplement: Supplementary file 1 [file SC-007-C5SC04155D-s001.pdf]

Electronic Supplementary Material (ESI) for Chemical Science.
This journal is © The Royal Society of Chemistry 2015

Electronic Supplementary Information

Achieving Remarkable Mechanochromism and White-Light Emission with Thermally Activated Delayed Fluorescence through Molecular Heredity Principle

Bingjia Xu,‡ Yingxiao Mu,‡ Zhu Mao, Zongliang Xie, Haozhong Wu, Yi Zhang,* Chongjun Jin, Zhenguo Chi,* Siwei Liu, Jiarui Xu, Yuan-Chun Wu, Po-Yen Lu, Alan Lien and Martin R. Bryce*

Experimental Section

Materials. Bis(4-fluorophenyl) sulfone, phenothiazine, potassium *tert*-butoxide (*t*-BuOK), 4-(9-*H*-carbozol-9-yl) phenylboronic acid, tetrakis(triphenylphosphine) palladium(0) and Aliquat 336 purchased from Alfa Aesar were used as received. Bis(4-bromo) sulfone (SBr_2)[1], 1-fluoro-4-((4-iodophenyl)sulfonyl)benzene (SFI)[2], 9,9'-(sulfonylbis([1,1'-biphenyl]-4',4-diyl))bis(9*H*-carbazole) (SC_2)[1] and 10,10'-(sulfonylbis(4,1-phenylene)) bis(10*H*-phenothiazine) (SP_2)[3] were synthesized according to the literature methods. Ultra-pure water was used in the experiments. All other reagents and solvents were purchased with analytical grade from Guangzhou Jincheng Company (China) and used without further purification. The water/tetrahydrofuran mixtures with different water fractions were prepared by slowly adding distilled water into the THF solution of the samples under ultrasonic at room temperature.

Measurements. Proton and carbon NMR (^{1}HNMR and ^{13}CNMR) spectra were measured on a superconducting Fourier transform nuclear magnetic (Bruker AVANCE 400) ($CDCl_3$ or DMSO-d_6, tetramethylsilane as the internal standard). The elemental analysis was performed with a Vario EL analyzer. The mass spectra were measured using thermospectrometers (DSQ & MAT95XP-HRMS). The UV-visible absorption spectra were determined on a Hitachi U-3900 spectrophotometer. The PL spectra were measured with a Shimadzu RF-5301 PC spectrometer or on an Ocean Optics Maya Pro2000 with a 310 nm Rhinospectrum RhinoLED as the excitation source. Wide-angle XRD measurements were performed at 293 K using a X-ray diffractometer (Smartlab, Rigaku Co.) with an X-ray source of Cu Kα (λ= 0.15406 nm) at 40 kV and 30 mA at a scan rate of 4° (2ϑ)/min. The fluorescence quantum yields of solid powders were measured in air on an integrating sphere (HAMAMATSU C11347) with a 330 nm UV light as the excitation source. The time-resolved emission decay behaviors were obtained using a spectrometer (FLSP980) from Edinburgh Instruments. The thermal behaviors were determined by differential scanning calorimetry (DSC) at heating rate of 10 ^{o}C/min under N_2 atmosphere using a NETZSCH thermal analyzer (DSC 204F1).The quantum chemistry calculation was performed at the B3LYP/6-31+G (d, p) level of theory using the DFT method in the Gaussian 09 software.

The single-crystal X-ray diffraction data for the compounds were collected from an Agilent Technologies Gemini A Ultra system with Cu-Kα radiation (λ=1.54178 Å) at 280(10) K or a Bruker Smart 1000 CCD with Cu-Kα radiation (λ =1.54178 Å) at 150(10) K. The structures were solved using direct methods following the difference Fourier syntheses. All non-hydrogen atoms were anisotropically refined through least-squares on F^2 using the SHELXTL program suite. The anisotropic thermal parameters were assigned to all non-hydrogen atoms. The hydrogen atoms attached to carbon were placed in idealized positions and refined using a riding model to the atom from which they were attached. The pictures of the three structures were produced using Diamond 3.2. CCDC 1421264, 1421265 and 1421266 contain the supplementary crystallographic data for this paper. These data can be obtained free of charge from The Cambridge Crystallographic Data Centre via www.ccdc.cam.ac.uk/data_request/cif.

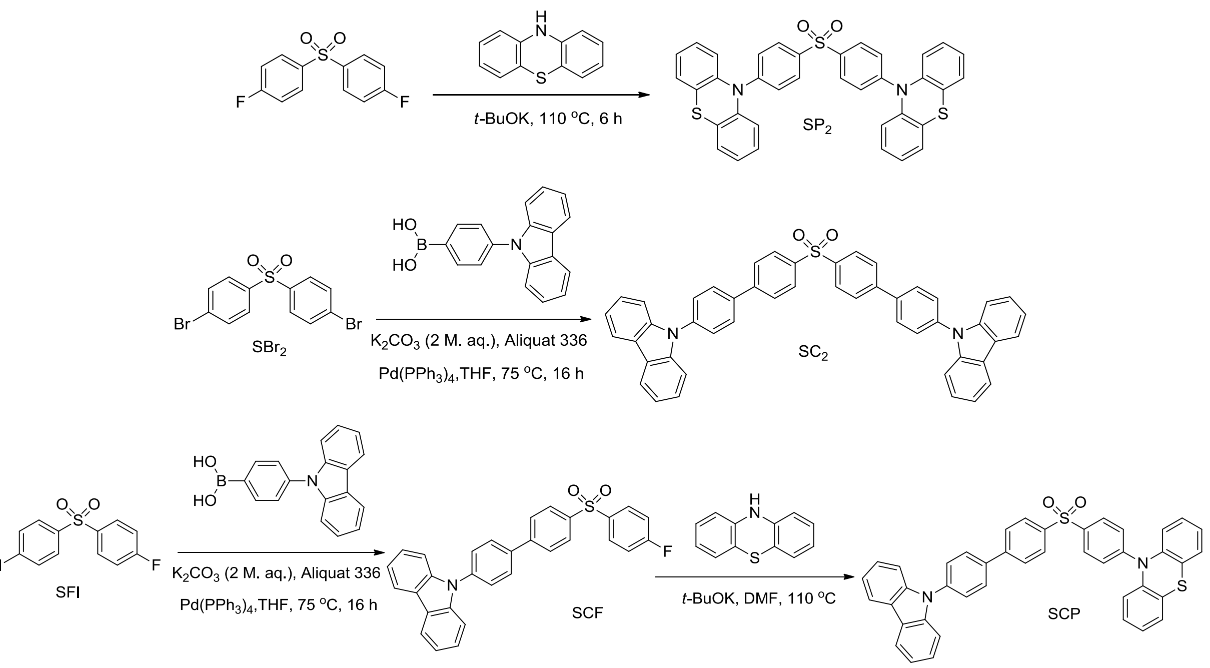

Scheme S1. Synthetic routes of the target compounds.

Synthesis of 10,10'-(sulfonylbis(4,1-phenylene))bis(10H-phenothiazine) (SP_2): Compound SP_2 was obtained as a light green solid by recrystallization from dichloromethane/*n*-hexane, following silica gel column chromatography. ^1H NMR (300 MHz, $CDCl_3$, δ): 7.80-7.72 (d, 4 H); 7.52-7.46 (d, 4 H); 7.39-7.21 (m, 12 H); 7.16-7.02 (d, 4 H). EIMS *m/z*: $[M]^+$ calcd for $C_{36}H_{24}N_2O_2S_3$, 612; found, 612. HRMS *m/z*: $[M]^+$ calcd for $C_{36}H_{24}N_2O_2S_3$, 612.1000; found, 612.0992. Anal. calcd for $C_{36}H_{24}N_2O_2S_3$: C 70.56, H 3.95, N 4.57, S 15.70; found: C 70.46, H 3.91, N 4.52, S 15.62. Single crystals for X-ray analysis were isolated by the slow solvent evaporation of a solution of SP_2 in a mixture of ethanol and dichloromethane (DCM).

Synthesis of 9,9'-(sulfonylbis([1,1'-biphenyl]-4',4-diyl))bis(9H-carbazole) (SC_2): Compound SC_2 was obtained as a white crystalline solid by recrystallizing from dichloromethane/*n*-hexane, following the silica gel column chromatography. ^1H NMR (300 MHz, DMSO-d_6, δ): 8.27-8.20 (d, 4 H); 8.19-8.11 (d, 4 H); 8.08-8.00 (t, 8 H); 7.80-7.74 (d, 4 H); 7.47-7.41 (m, 8 H), 7.33-7.26 (td, 4 H). EIMS *m/z*: $[M]^+$ calcd for $C_{48}H_{32}N_2O_2S$, 700; found, 700. HRMS *m/z*: $[M]^+$ calcd for $C_{48}H_{32}N_2O_2S$, 700.2184; found, 700.2174. Anal. calcd for $C_{48}H_{32}N_2O_2S$: C 82.26, H 4.60, N 4.00, S 4.58; found: C 82.18, H 4.66, N 4.04, S 4.51. Single crystals for X-ray analysis were isolated by the slow solvent evaporation of a solution of SC_2 in a mixture of ethanol and DCM.

Synthesis of 9-(4'-((4-fluorophenyl)sulfonyl)-[1,1'-biphenyl]-4-yl)-9H-carbazole (SCF):1-fluoro-4-((4-iodophenyl) sulfonyl)benzene (SFI) (1.00 g, 2.76 mmol) and (4-(9*H*-carbazol-9-yl)phenyl)boronic acid (0.99, 3.45 mmol) were dissolved in tetrahydrofuran (THF) (30 mL), and then 2 M. aqueous K_2CO_3 solution (3.5 ml) was added. The mixture was stirred for 40 min under an argon atmosphere at room temperature. Then $Pd(PPh_3)_4$ catalyst was added, and the reaction mixture was stirred at 75 ˚C for 16 h. After cooling to room temperature, the product was concentrated and purified by silica gel column chromatography with dichloromethane/*n*-hexane (v/v=4:5) as eluent. Compound SCF was obtained as a white solid in 81% yield (1.07 g). ^1H NMR (400 MHz, $CDCl_3$, δ): 8.19-8.14 (d, 2 H); 8.19-8.00 (m, 4 H); 7.84-7.77 (m, 4 H); 7.72-7.66 (d, 2 H); 7.49-7.40 (m, 4 H), 7.35-7.28 (t, 2 H) , 7.26-7.18 (t, 2 H). ^{13}C NMR (100 MHz,

$CDCl_3$, δ): 168.17, 165.63, 146.69, 142.02, 141.80, 139.68, 139.33, 139.17, 129.68, 129.37, 128.87, 127.44, 124.98, 121.63, 118.16, 117.93, 111.37, 111.08. EIMS *m/z*: $[M]^+$ calcd for $C_{30}H_{20}FNO_2S$, 477; found, 477. HRMS *m/z*: $[M]^+$ calcd for $C_{30}H_{20}FNO_2S$, 477.1199; found, 477.1196. Anal. calcd for $C_{30}H_{20}FNO_2S$: C 75.45, H 4.22, N 2.93, S 6.71; found: C 75.49, H 4.17, N 2.96, S 6.75.

Synthesis of 10-(4-((4'-(9H-carbazol-9-yl)-[1,1'-biphenyl]-4-yl)sulfonyl)phenyl)-10H-phenothiazine (SCP): To a solution of phenothiazine (0.52 g, 2.62 mmol) in dry *N*,*N*-dimethylformamide (DMF) (20 ml) potassium *tert*-butoxide (*t*-BuOK) (0.59 g, 5.24 mmol) was added. After the solution was stirred at room temperature under an argon atmosphere for 20 min, SCF (1.00 g, 2.09 mmol) was added, and then the mixture was stirred at 110 ˚C for an additional 6 h. After cooling, the mixture was poured to 200 ml of ice water, and the precipitate was filtered and washed with water for several times. The crude product was then recrystallized from dichloromethane/acetone to produce a white crystalline solid in 62% yield (0.86g).^{1}H NMR (300 MHz, $CDCl_3$, δ): 8.19-8.11 (d, 2 H); 8.06-7.99 (d, 2 H); 7.85-7.71 (m, 6 H); 7.70-7.64 (d, 2 H); 7.50-7.37 (dd, 6 H), 7.35-7.26 (m, 6 H), 7.23-7.17 (m, 2 H), 7.14-7.09 (d, 2 H). ^{13}C NMR (100 MHz, $CDCl_3$, δ): 150.94, 146.15, 142.75, 142.37, 142.05, 139.59, 139.50, 134.68, 134.59, 130.14, 129.20, 128.84, 128.74, 127.59, 127.40, 124.94, 121.75, 121.57, 117.77, 111.36, 111.10. EIMS *m/z*: $[M]^+$ calcd for $C_{42}H_{28}N_2O_2S_2$, 656; found, 656. HRMS *m/z*: $[M]^+$ calcd for $C_{42}H_{28}N_2O_2S_2$, 656.1592; found, 656.1584. Anal. calcd for $C_{42}H_{28}N_2O_2S_2$: C 76.80, H 4.30, N 4.27, S 9.76; found: C 76.75, H 4.34, N 4.31, S 9.70. Single crystals for X-ray analysis were isolated by the slow solvent evaporation of a solution of SCP in a mixture of ethanol and DCM.

Figures

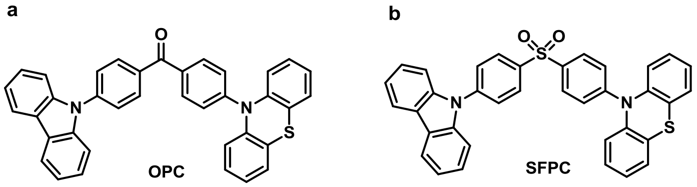

Fig. S1 Molecular structure of OPC and SFPC.[4]

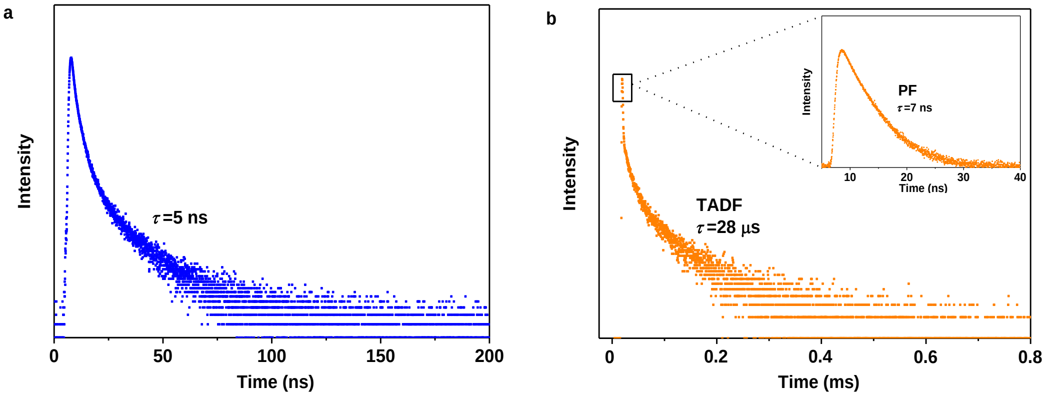

Fig. S2 Emission decays curves. (a) Pristine SC_2. (b) Pristine SP_2.

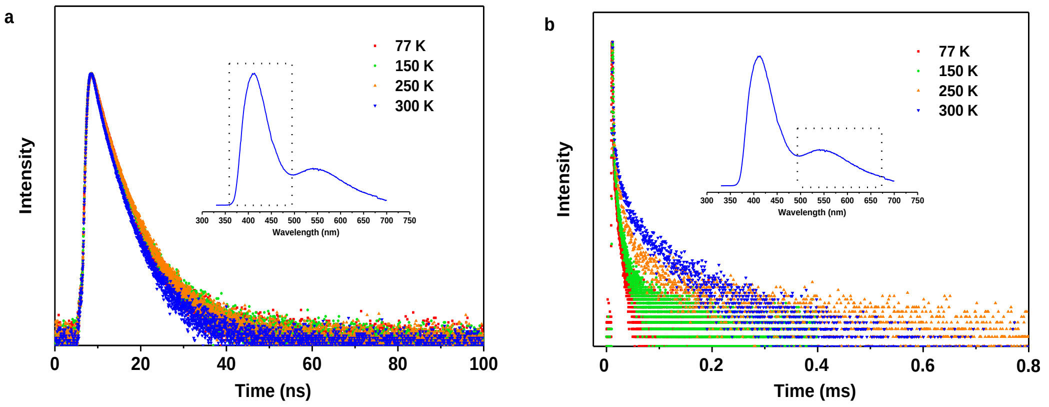

Fig. S3 Emission decays of solid powders for SCP at different temperature. (a) for the emission in blue-light region; (b) for the emission in yellow-light region.

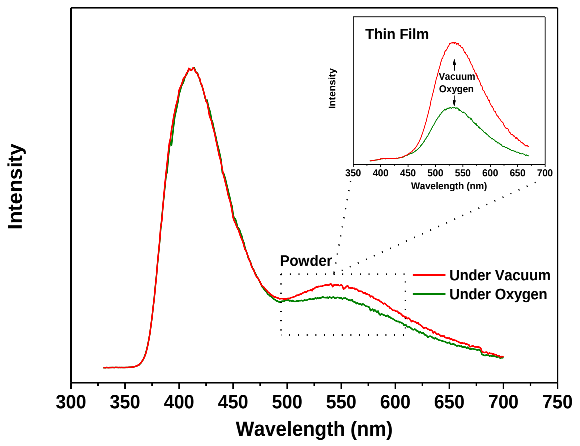

Fig. S4 PL spectra of compound SCP in solid powder and thin film (inset) under different atmospheres.

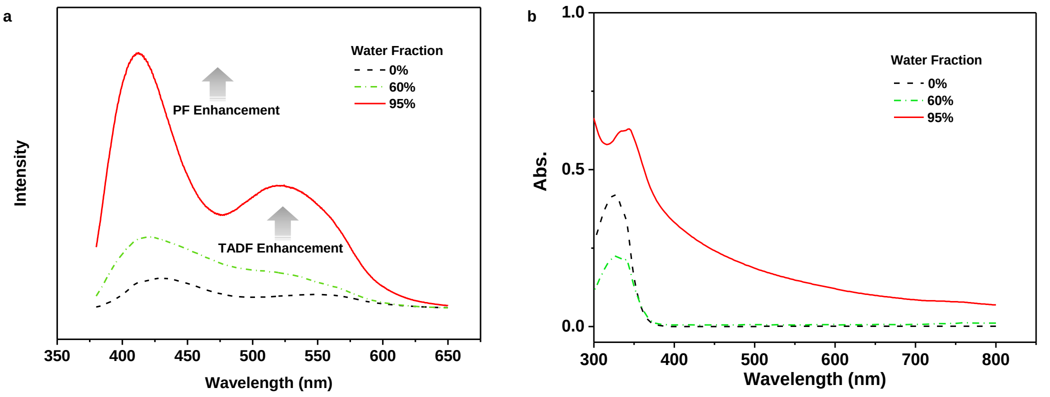

Fig. S5 Photophysical properties of SCP in THF/water with different water fractions. (a) PL spectra. (b) UV-visible absorption spectra.

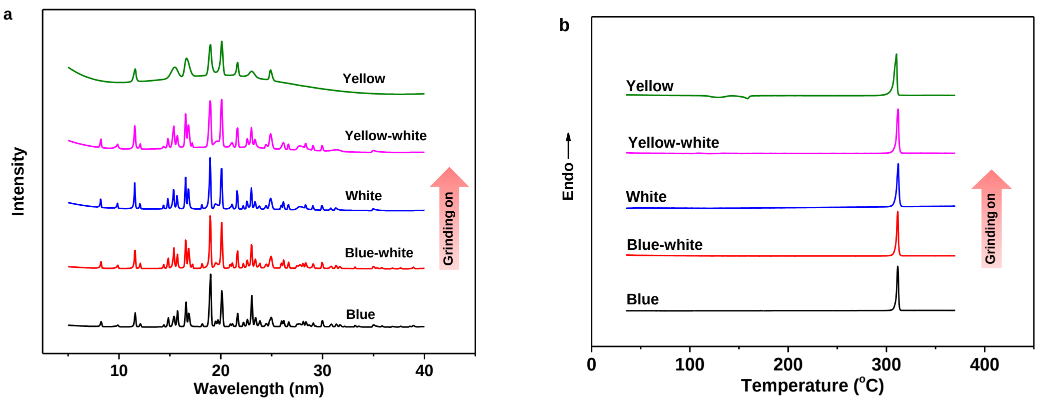

Fig. S6 Morphological and thermal properties of SCP under grinding. (a) XRD patterns. (b) DSC curves.

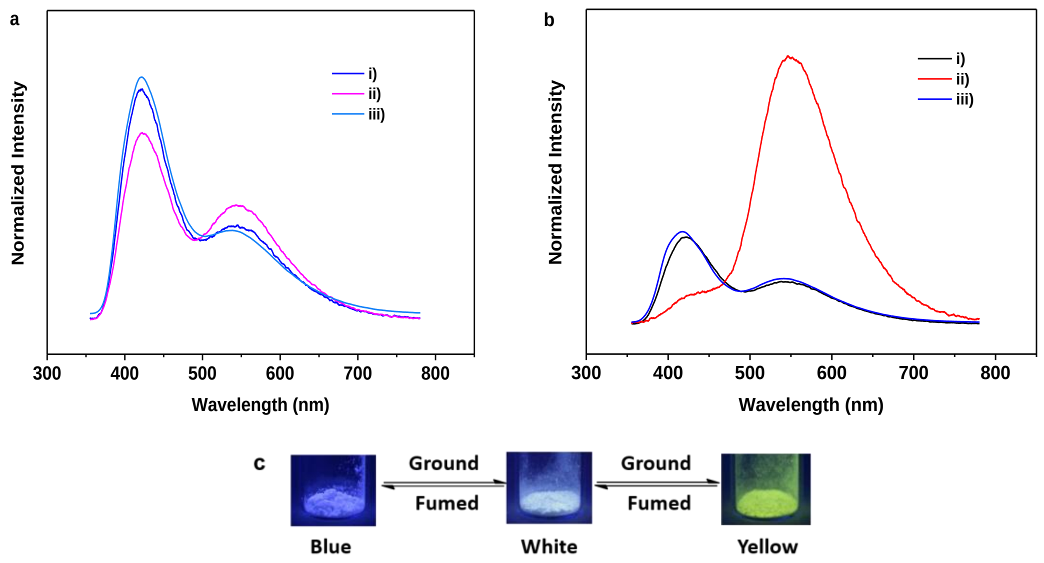

Fig. S7 Changes of PL spectra and fluorescent colors of SCP by applying mechanical force or fuming with dichloromethane (DCM) vapor. (a) switch between the blue- and white-emission: i) pristine blue-light powder ii) white-emitting powder obtained from pristine sample by grinding; iii) blue-light powder obtained from the white-light sample by fuming with DCM vapor for about 30 min. (b) switch between the white- and yellow-emission: i) white-emitting powder obtained from the pristine blue-light sample by grinding; ii) yellow-emitting powder obtained from the white-light sample by further grinding; iii) white-emitting powder obtained from the yellow-light powder by fuming with DCM vapor for about 20 min. (c) fluorescent images of SCP in different colors under the illumination of 365 nm UV light.

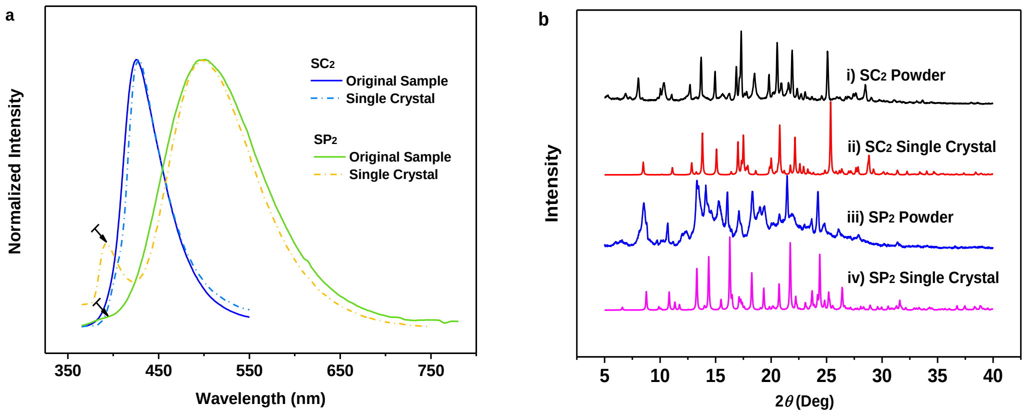

Fig. S8 Photophysical and morphological properties of the original powders and single crystals of SC_2 and SP_2. (a) Experimental PL spectra. (b) XRD patterns: i) experimental XRD pattern of SC_2 powder; ii) simulated XRD pattern of SC_2 from single crystal data; iii) experimental XRD pattern of SP_2 powder; iv) simulated XRD pattern of SP_2 from single crystal data.

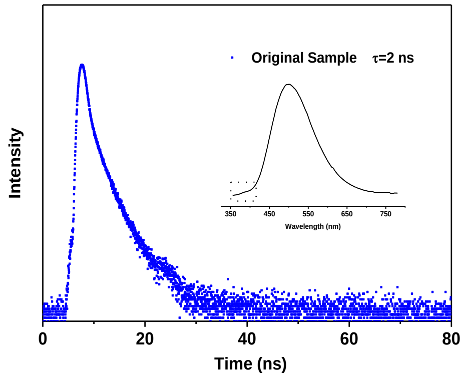

Fig. S9 Emission decay curve of SP_2 in solid state at room temperature.

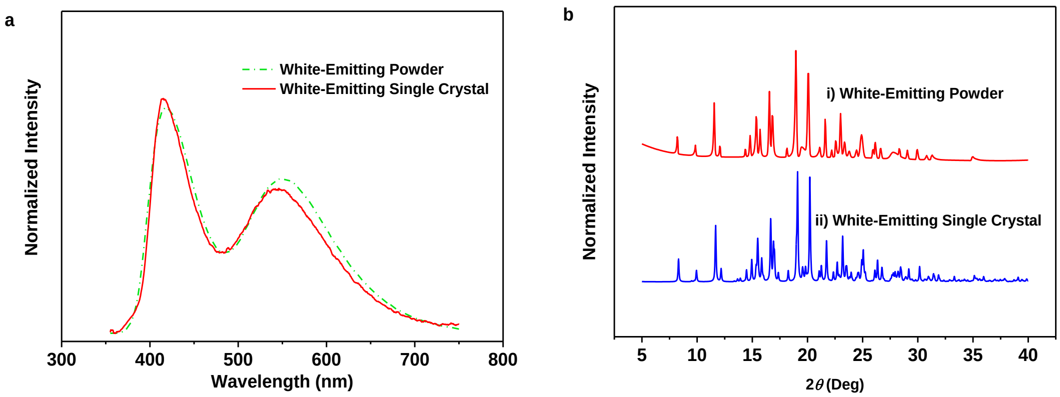

Fig. S10 PL spectra and XRD patterns of the white-emitting powder and the white-emitting single crystals of SCP. (a) Experimental PL spectra. (b) XRD patterns: i) experimental XRD pattern of SCP powder; ii) simulated XRD pattern of SCP from single crystal data.

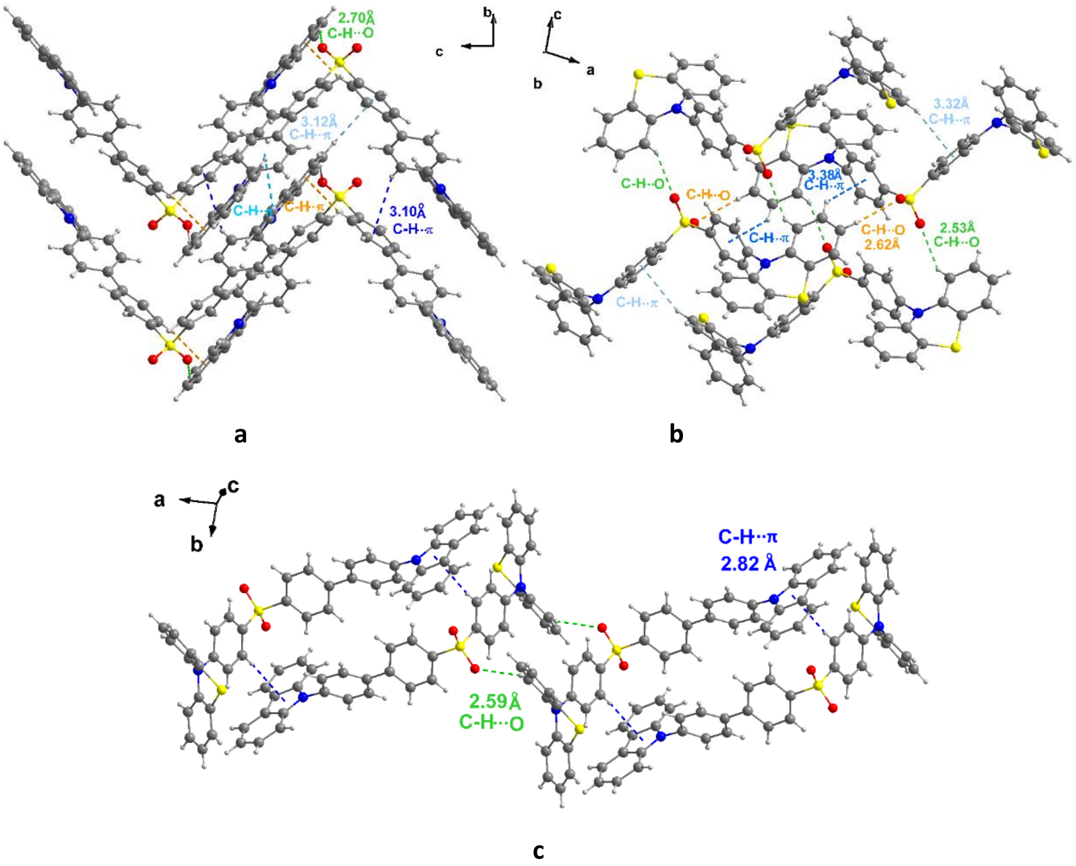

Fig. S11 Molecular packing and intermolecular interactions of the compounds in single crystals. (a) SC_2. (b) SP_2. (c) SCP.

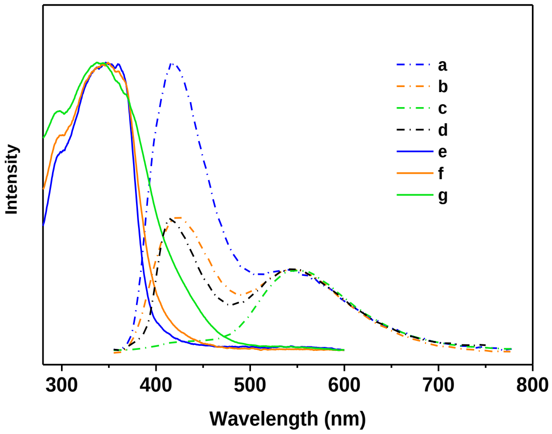

Fig. S12 UV-visible absorption and PL spectra of SCP: (a) PL of blue powder; (b) PL of white powder; (c) PL of yellow powder; (d) PL of white-emitting single crystal; (e) UV of blue powder; (f) UV of white powder; (g) UV of yellow powder.

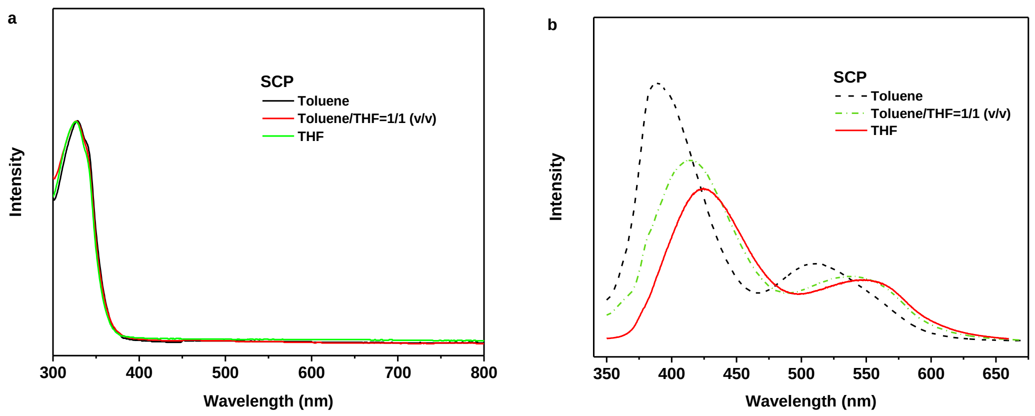

Fig. S13 Photophysical properties of SCP in different solvents. (a) UV-visible absorption spectra. (b) PL spectra.

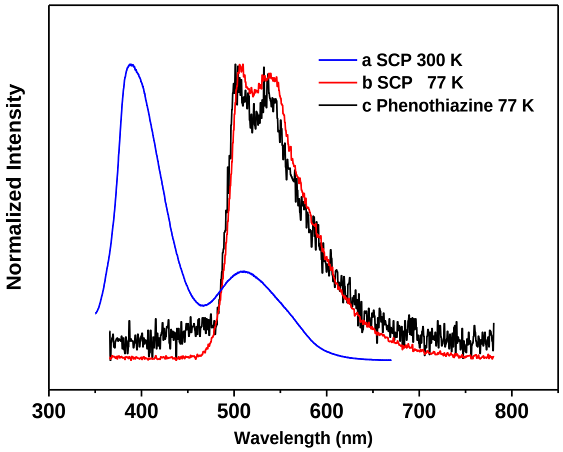

Fig. S14 Photophysical properties of the compounds. (a) fluorescence spectrum of SCP in toluene at 300 K. (b) phosphorescence spectra of SCP in toluene at 77 K (measured with 16 ms delay). (c) phenothiazine in toluene at 77 K (measured with 16 ms delay).

NMR and Mass Spectra

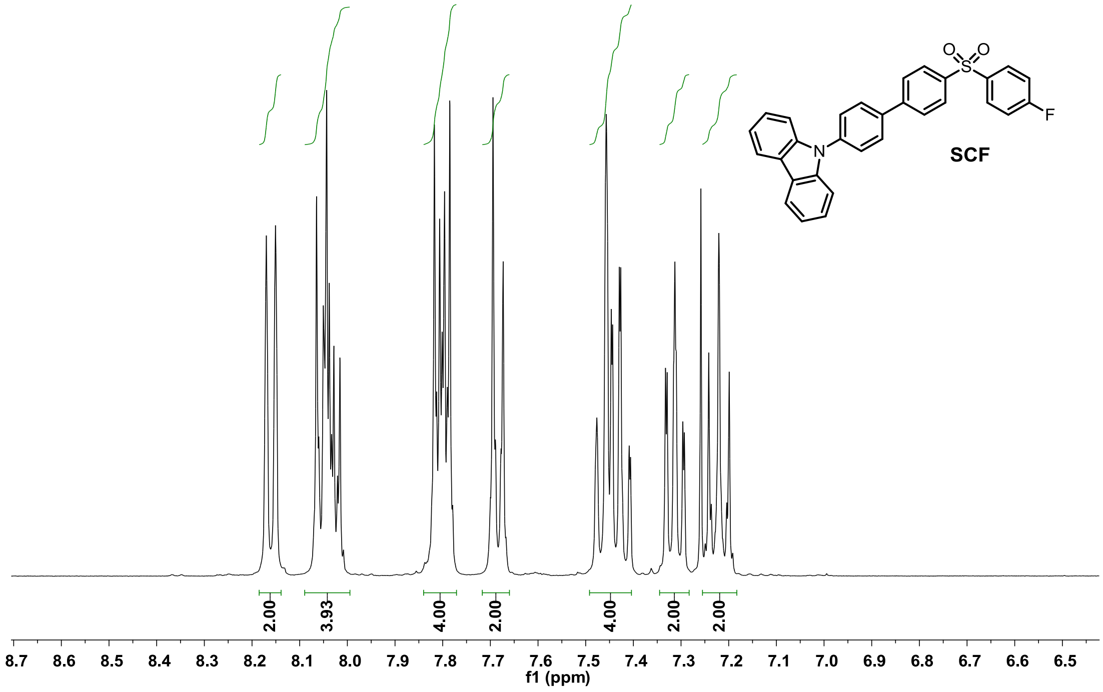

Fig. S15 ^{1}H NMR spectrum of SCF in $CDCl_3$.

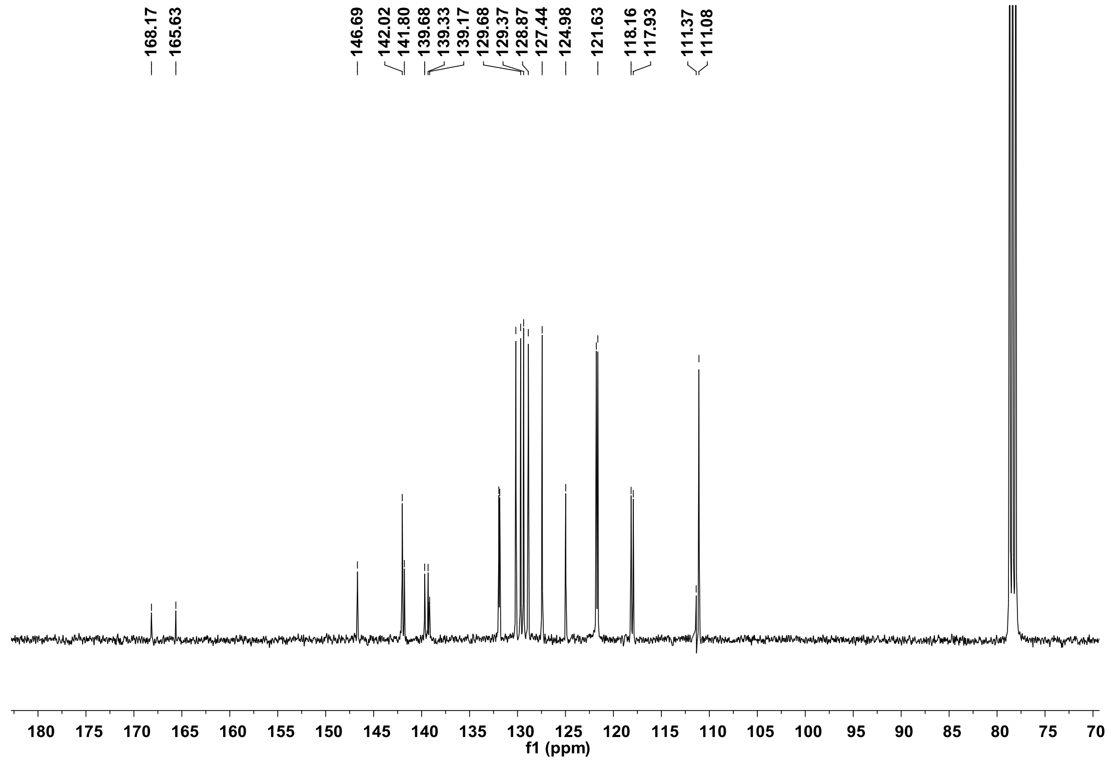

Fig. S16 ^{13}C NMR spectrum of SCF in $CDCl_3$.

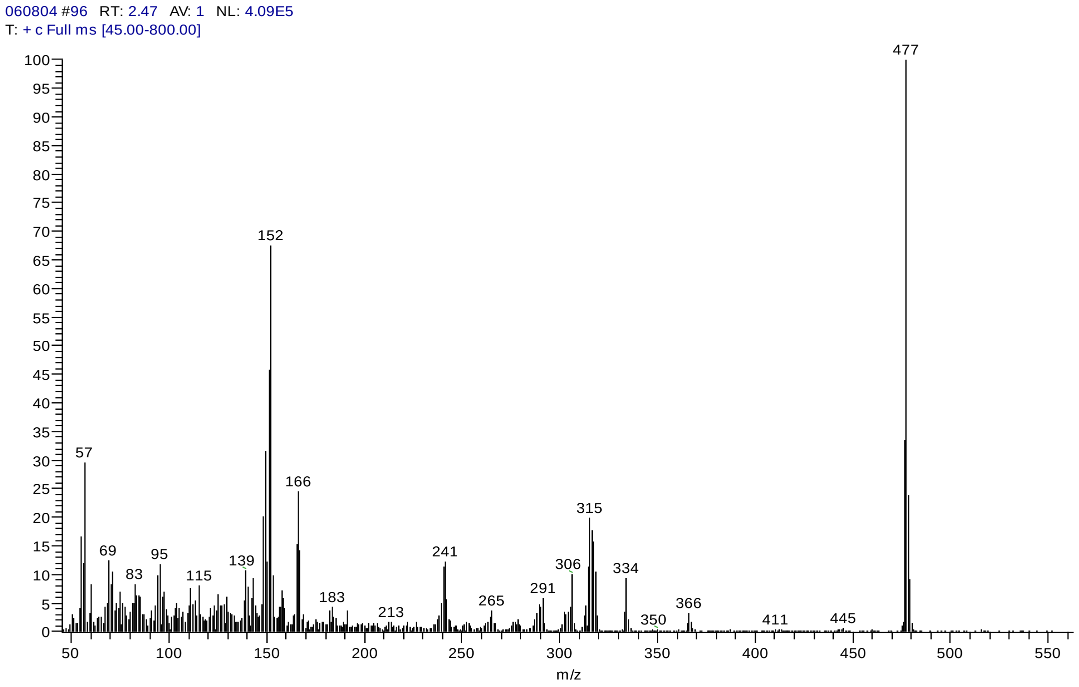

Fig. S17 EI-MS of SCF.

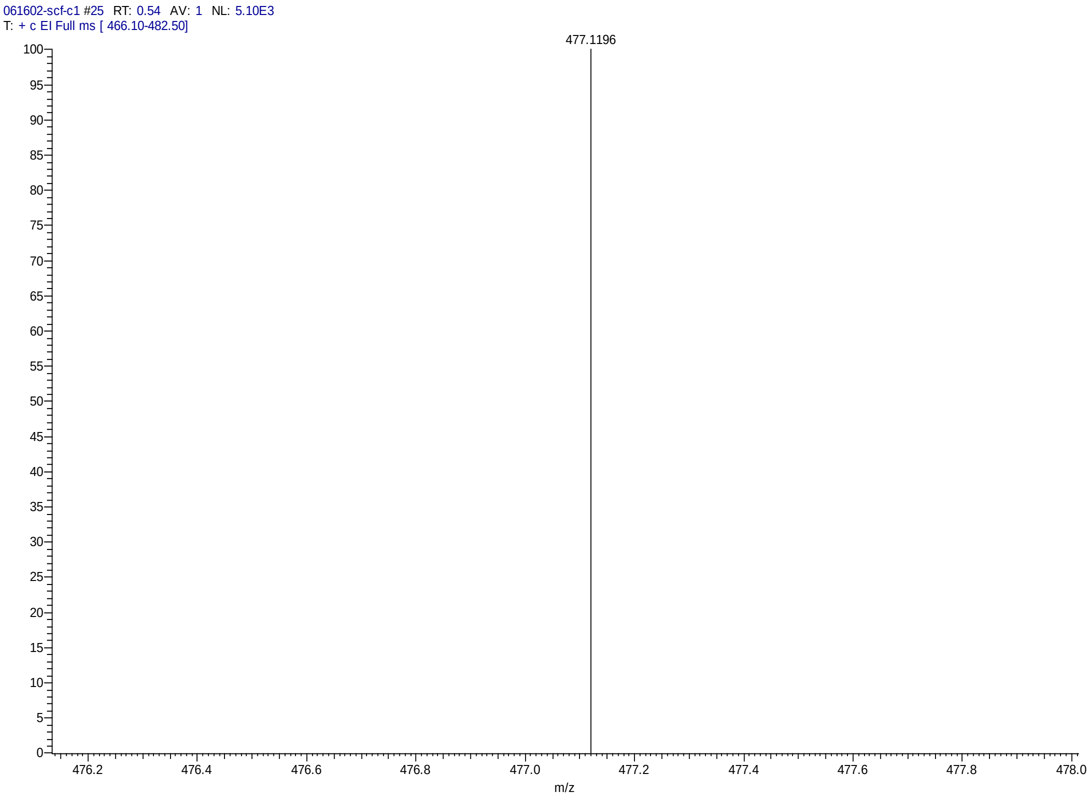

Fig. S18 HRMS of SCF.

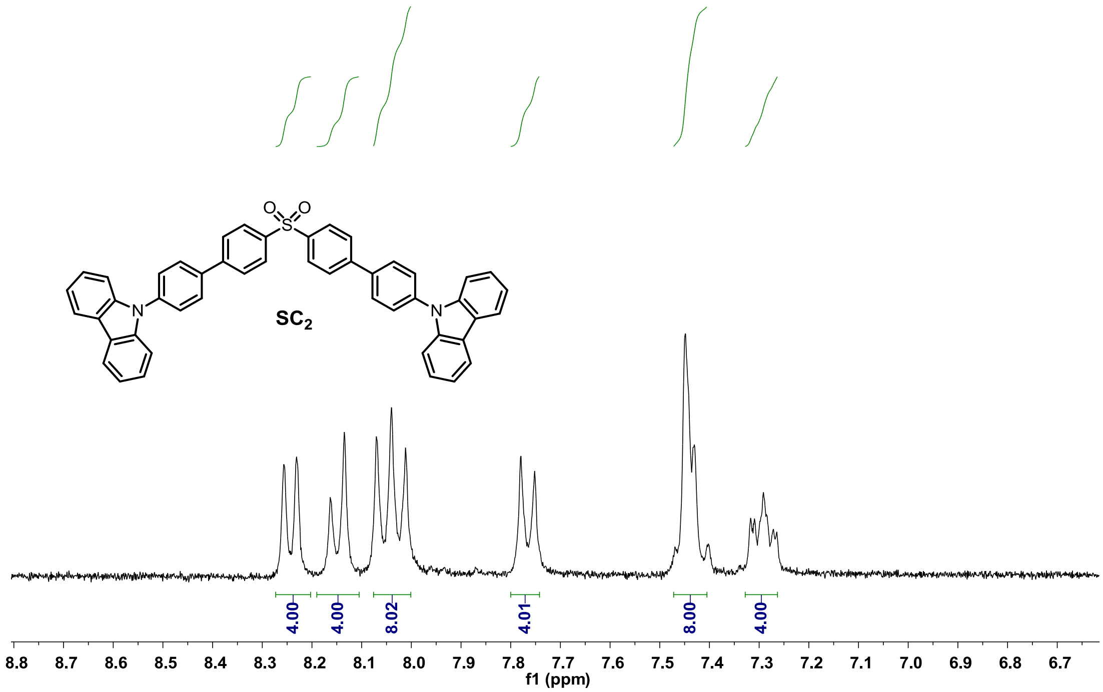

Fig. S19 ^{1}H NMR spectrum of SC$_2$ in DMSO-d_6.

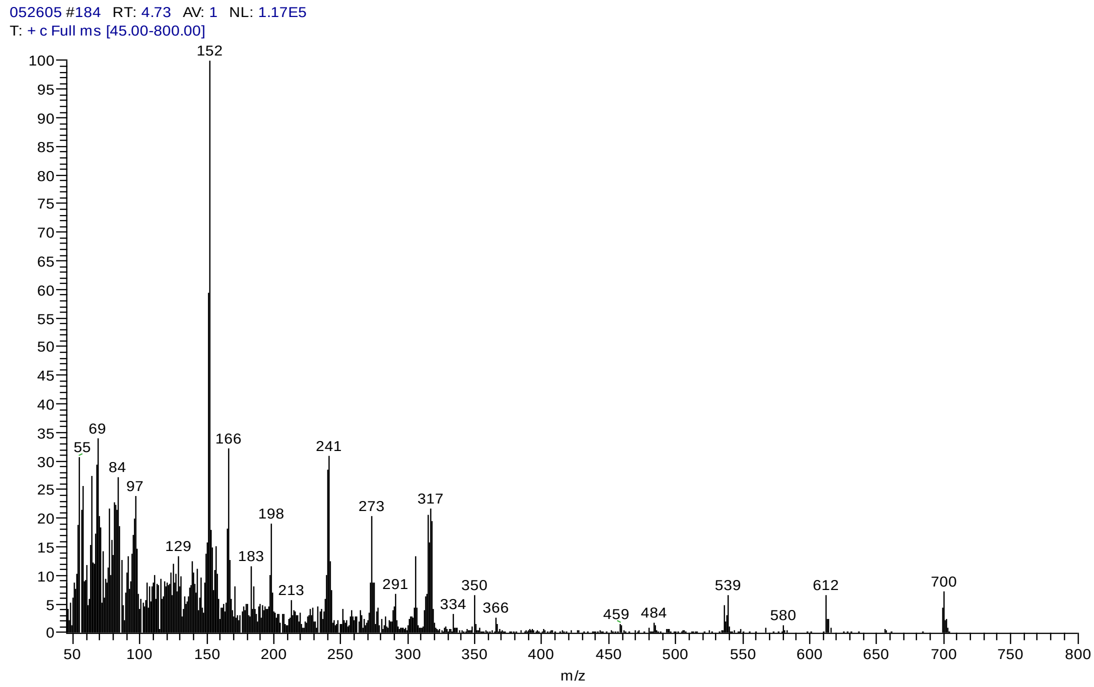

Fig. S20 EI-MS of SC$_2$.

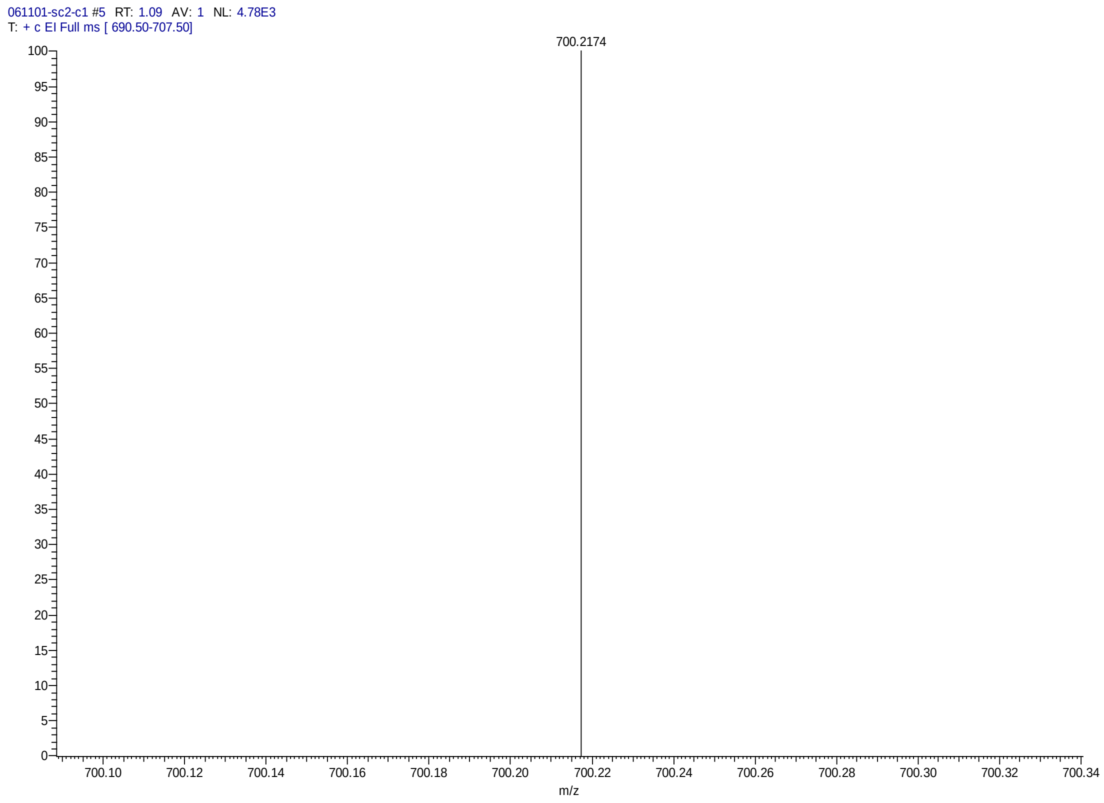

Fig. S21 HRMS of SC_2.

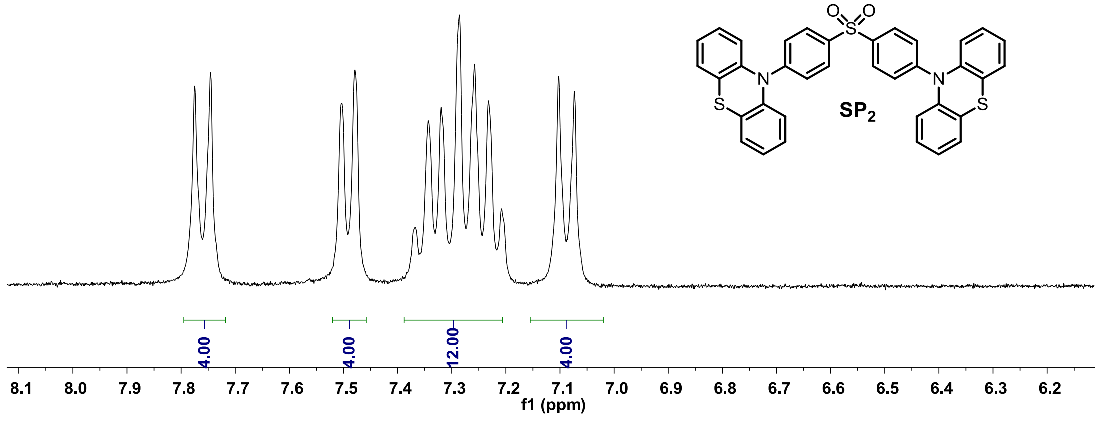

Fig. S22 ^{1}H NMR spectrum of SP_2 in $CDCl_3$.

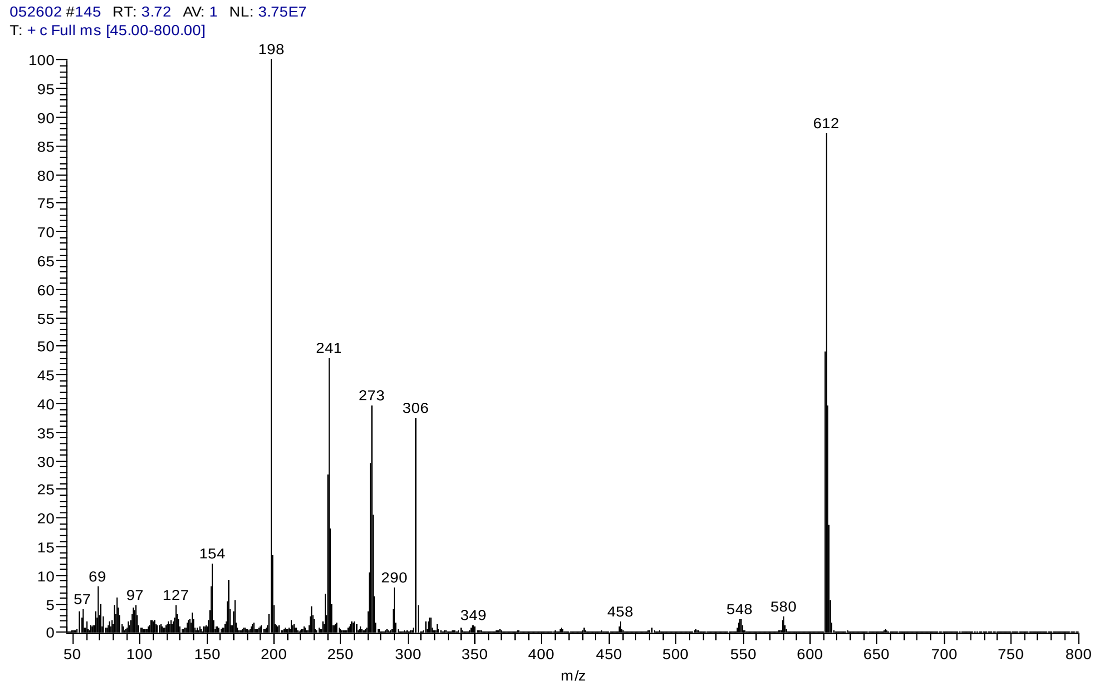

Fig. S23 EI-MS of SP_2.

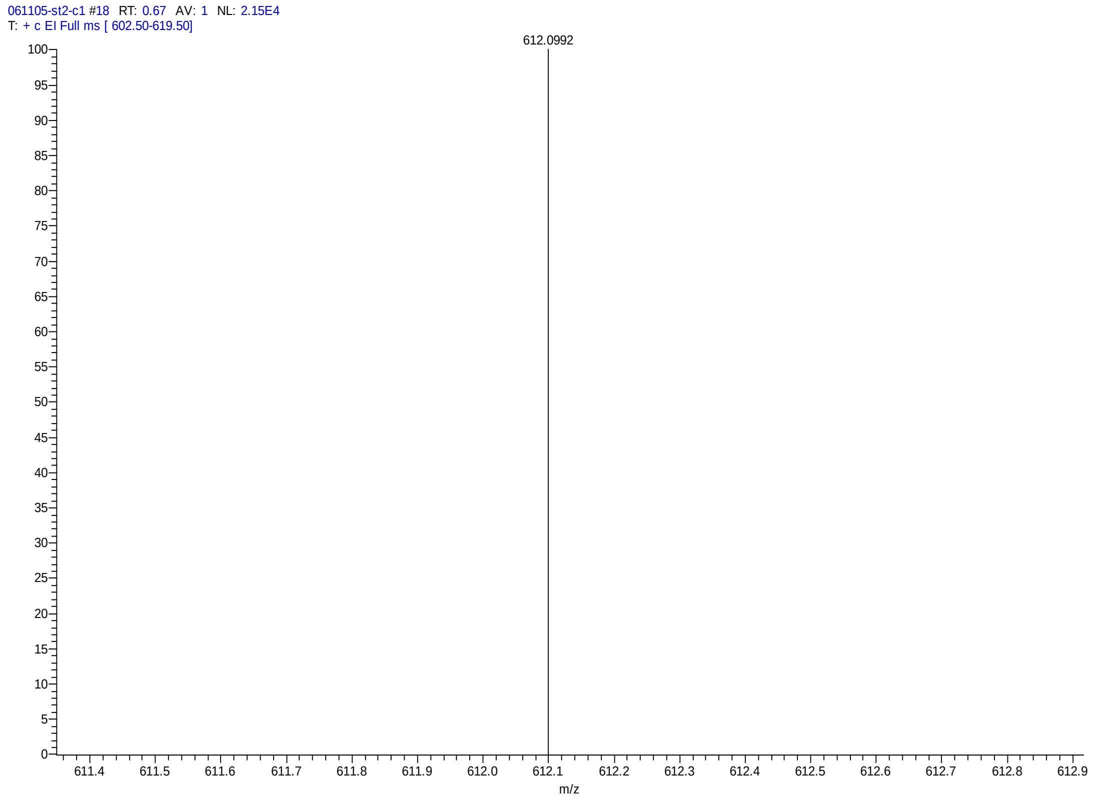

Fig. S24 HRMS of SP_2.

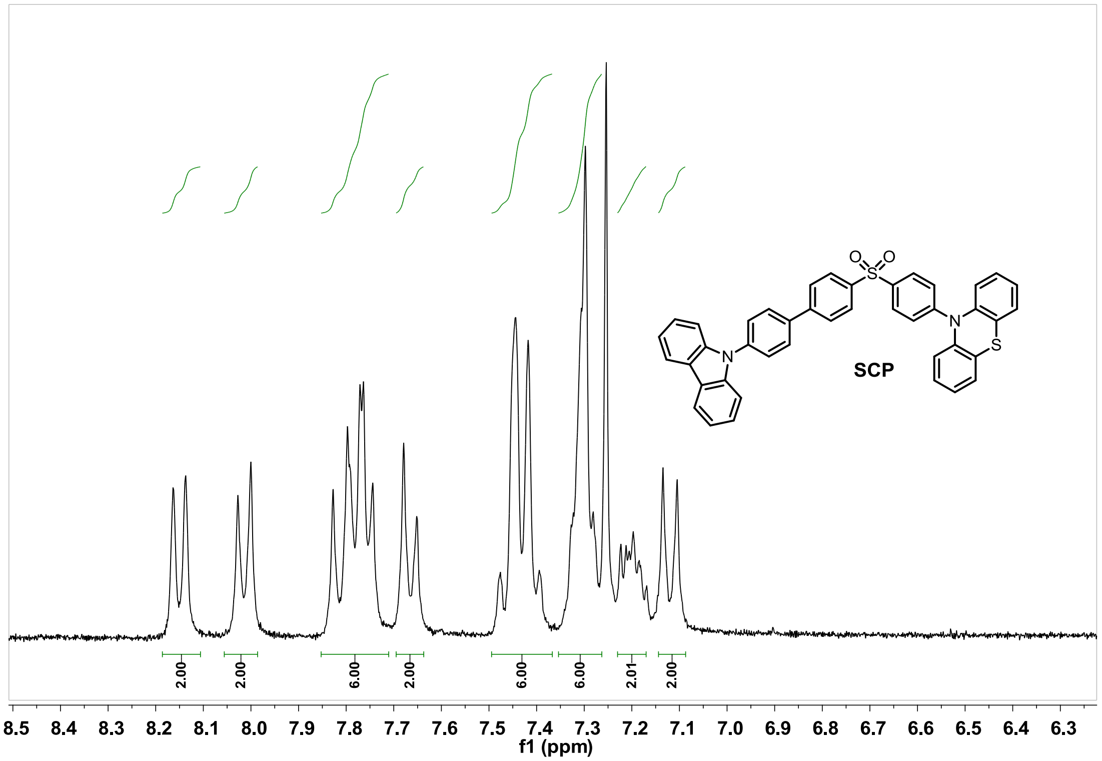

Fig. S25 ^{1}H NMR spectrum of SCP in $CDCl_3$.

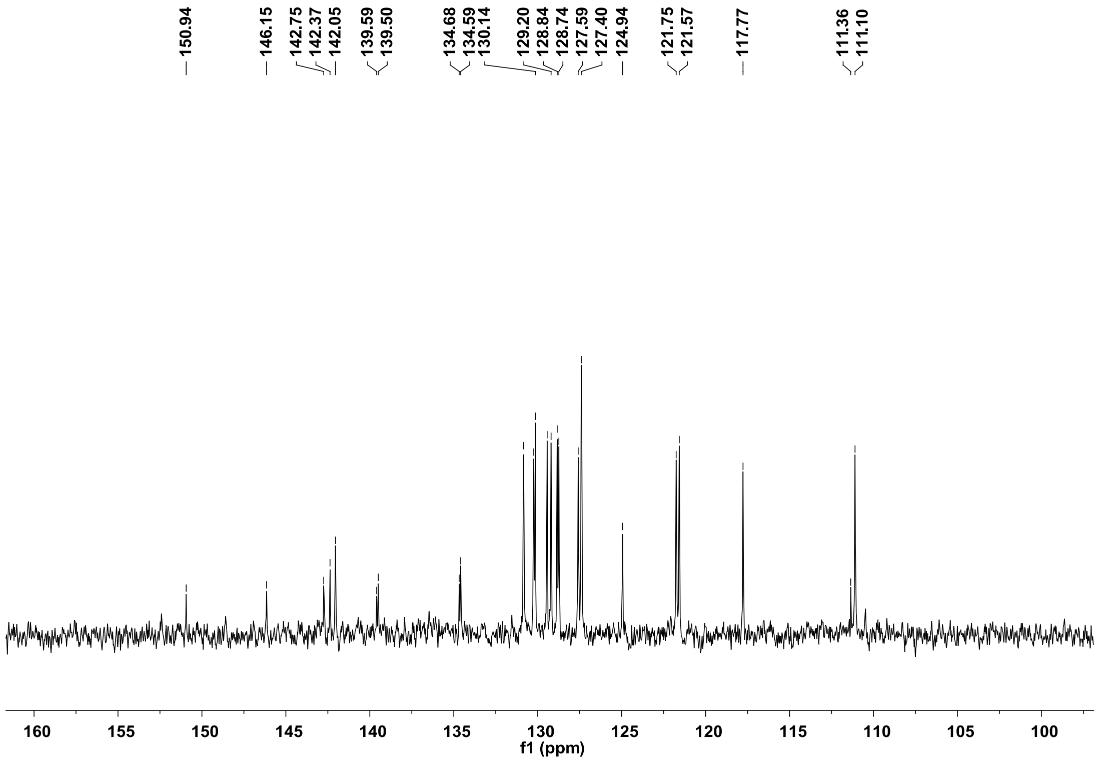

Fig. S26 ^{13}C NMR spectrum of SCP in $CDCl_3$.

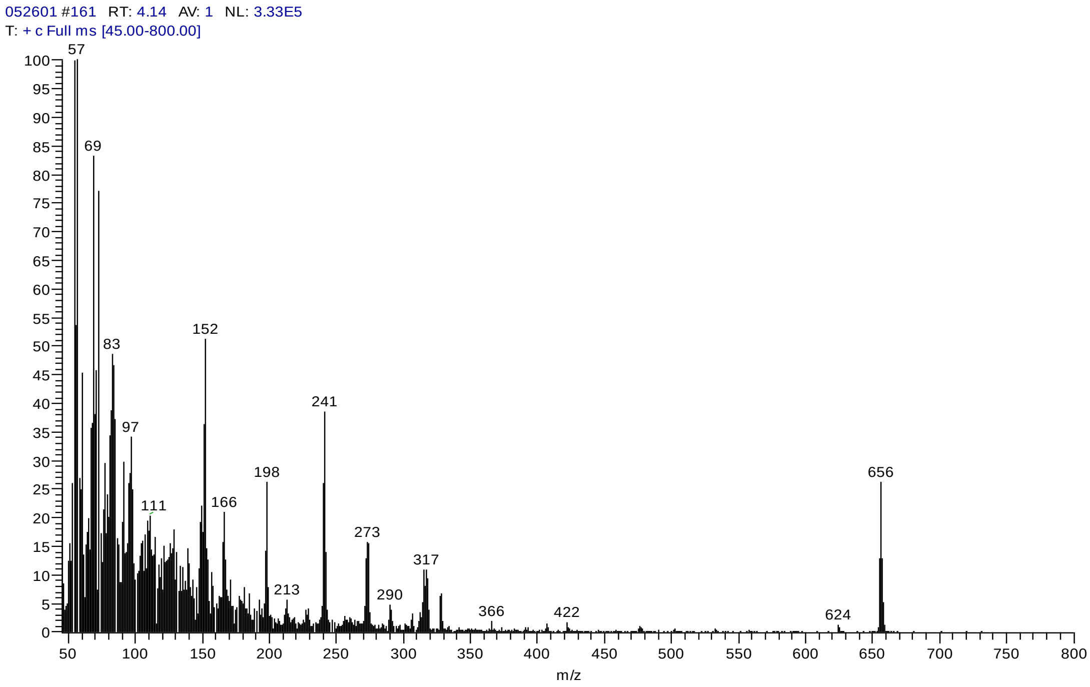

Fig. S27 EI-MS of SCP.

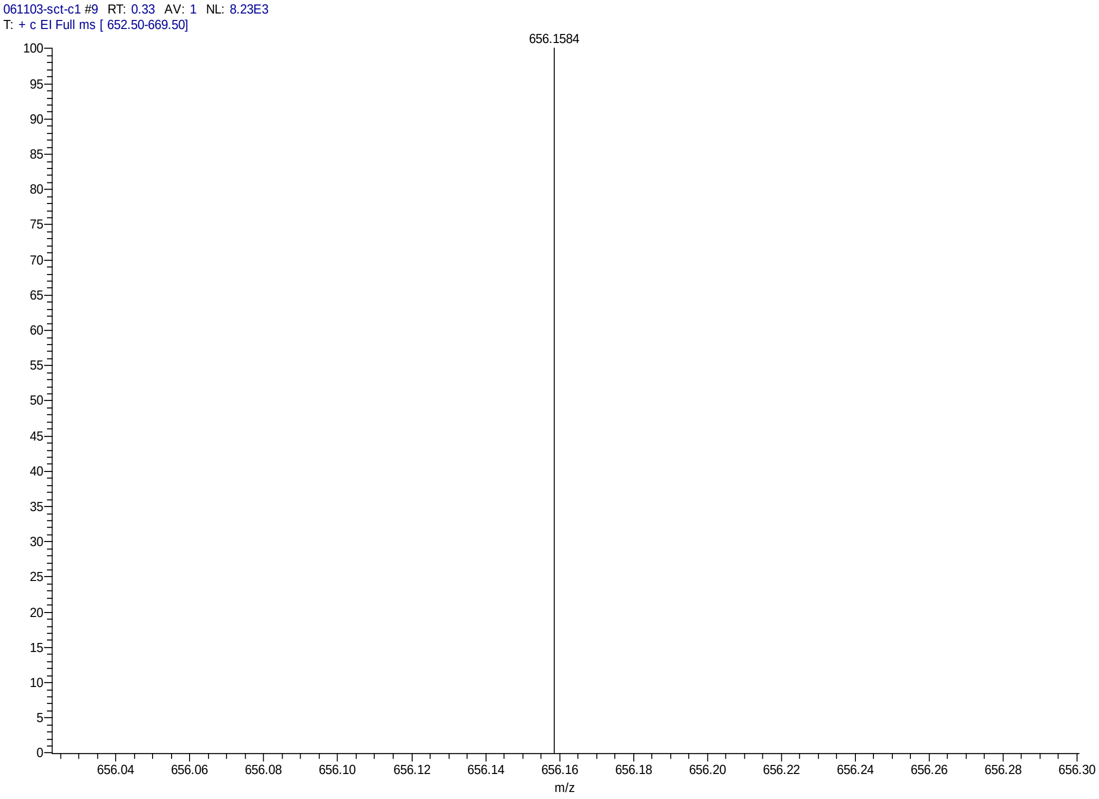

Fig. S28 HRMS of SCP.

Reference

1 J. Ye, Z. Chen, M. K. Fung, C. Zheng, X. Ou, X. Zhang, Y. Yuan and C. S. Lee, *Chem. Mater.*, 2013, **25**, 2630.

2 Z. Mao, Z. Yang, Y. Mu, Y. Zhang, Y. F. Wang, Z. Chi, C. C. Lo, S. Liu, A. Lien and J. Xu, *Angew. Chem. Int. Ed.*, 2015, **54**, 6270.

3 S. Xu, T. Liu, Y. Mu, Y. F. Wang, Z. Chi, C. C. Lo, S. Liu, Y. Zhang, A. Lien and J. Xu, *Angew. Chem. Int. Ed.*, 2015, **54**, 874.

4 Z. Xie, C. Chen, S. Xu, J. Li, Y. Zhang, S. Liu, J. Xu and Z. Chi, *Angew. Chem. Int. Ed.*, 2015, **54**, 7181.
